# Genetic Testing of Breast Cancer Patients with Very Early-Onset Breast Cancer (≤30 Years) Yields a High Rate of Germline Pathogenic Variants, Mainly in the BRCA1, TP53, and BRCA2 Genes

**DOI:** 10.3390/cancers16132368

**Published:** 2024-06-27

**Authors:** Paraskevi Apostolou, Vasiliki Dellatola, Athanasios Papathanasiou, Despoina Kalfakakou, Elena Fountzilas, Dimitrios Tryfonopoulos, Sofia Karageorgopoulou, Drakoulis Yannoukakos, Irene Konstantopoulou, Florentia Fostira

**Affiliations:** 1 Human Molecular Genetics Laboratory, National Center for Scientific Research “Demokritos”, 15341 Athens, Greece; apostoloup@rrp.demokritos.gr (P.A.); v.dellatola@rrp.demokritos.gr (V.D.); t.papathanasiou@rrp.demokritos.gr (A.P.); yannouka@rrp.demokritos.gr (D.Y.); reena@rrp.demokritos.gr (I.K.); 2 Perlmutter Cancer Center, NYU Langone Health, New York, NY 10016, USA; despoina.kalfakakou@nyulangone.org; 3 Department of Medical Oncology, St. Lukes’s Hospital, 55236 Thessaloniki, Greece; fountzila@oncogenome.gr; 4 European University Cyprus, 6, Diogenes 2404 Engomi, Nicosia 1516, Cyprus; 5 Department of Medical Oncology, “Agios Savvas” Anticancer Hospital, 11522 Athens, Greece; oncology2@agsavvas-hosp.gr; 6 Third Department of Medical Oncology, IASO Clinic, 15123 Athens, Greece; skarageorgopoulou@iaso.gr

**Keywords:** hereditary breast cancer, *BRCA1*, *BRCA2*, TP53, early onset, genetic testing, survival

## Abstract

**Simple Summary:**

Identification of germline pathogenic variants in breast cancer patients holds significant importance for accurate risk assessment and therapeutic interventions. However, research focusing on very young breast cancer patients remains limited. Our objective was to describe the prevalence, gene, and variant spectra alongside clinicopathological characteristics and outcomes of women diagnosed with breast cancer ≤30 years. Our observations revealed that one in three patients carried predisposing variants distributed in eight established breast cancer genes. Predominantly, causative variants implicated loss-of-function of *BRCA1*, *TP53*, and *BRCA2*, with *TP53* emerging as the second most frequently mutated gene within this cohort. While carrier status did not impact event-free survival, carriers who underwent neoadjuvant chemotherapy exhibited improved prognostic outcomes. Our data underscore the substantial proportion of patients with hereditary predisposition, advocate for the inclusion of *TP53* genetic testing, and suggest possible benefits of neoadjuvant chemotherapy in this very young group of breast cancer patients.

**Abstract:**

Early-onset breast cancer constitutes a major criterion for genetic testing referral. Nevertheless, studies focusing on breast cancer patients (≤30 years) are limited. We investigated the contribution and spectrum of known breast-cancer-associated genes in 267 Greek women with breast cancer ≤30 years while monitoring their clinicopathological characteristics and outcomes. In this cohort, a significant proportion (39.7%) carried germline pathogenic variants (PVs) distributed in 8 genes. The majority, namely 36.7%, involved *BRCA1*, *TP53,* and *BRCA2*. PVs in *BRCA1* were the most prevalent (28.1%), followed by *TP53* (4.5%) and BRCA2 (4.1%) PVs. The contribution of PVs in *CHEK2*, *ATM*, *PALB2*, *PTEN*, and *RAD51C* was limited to 3%. In the patient group ≤26 years, *TP53* PVs were significantly higher compared to the group 26–30 years (*p* = 0.0023). A total of 74.8% of *TP53* carriers did not report a family history of cancer. Carriers of PVs receiving neoadjuvant chemotherapy showed an improved event-free survival (*p* < 0.0001) compared to non-carriers. Overall, many women with early-onset breast cancer carry clinically actionable variants, mainly in the *BRCA1/2* and *TP53* genes. The inclusion of timely testing of *TP53* in these patients provides essential information for appropriate clinical management. This is important for countries where reimbursement involves the cost of genetic analysis of *BRCA1/2* only.

## 1. Introduction

Breast cancer diagnosis at an early age, generally before the age of 40 years, is a typical indicator of hereditary cancer predisposition. A significant proportion of young patients, even in the absence of family history, will carry pathogenic variants in the *BRCA1* and *BRCA2* genes [1]. Clinical implementation of *BRCA1* and *BRCA2* genetic testing allows appropriate risk management and decision-making through tailored surveillance protocols and options for risk-reducing interventions in a timely manner [2], therefore improving both patient outcomes and survival.

Moving beyond *BRCA1* and *BRCA2*, rare germline pathogenic variants (PVs) in other genes that have a role in the DNA repair pathway of homologous recombination also confer risks for breast cancer. Specifically, PVs in *PALB2, ATM, CHEK2, RAD51C,* and *RAD51D* are associated with increased lifetime breast cancer risk, ranging from 20% to 58%, depending on the defective gene and identified variant, while influenced by family history [3,4,5,6,7]. More uncommonly, highly penetrant PVs in *CDH1, PTEN, STK11,* and *TP53* are associated with a very high risk of breast cancer diagnosis. Loss of function in these genes is known to be associated with rare cancer syndromes characterized by a wide range of clinical manifestations, including breast cancer [8,9]. Specifically, PVs in *TP53* are known to cause Li–Fraumeni syndrome, which is associated with numerous malignancies, even in childhood. These include sarcomas, adrenocortical carcinomas, brain tumors, leukemias, and breast cancer. Female *TP53* carriers have a significant risk of developing breast cancer, which can be as high as 85% by the age of 60 years [10,11], with the majority of breast cancer diagnoses being human epidermal growth factor receptor-2 (HER2) positive [12,13,14]. *TP53* carriers are at high risk of developing breast cancer at a very young onset, and their options for risk-reduction strategies have to be discussed and taken into consideration. Avoidance of radiation is strongly suggested in these individuals due to their increased radiosensitivity and subsequent risk of developing new malignancies [15,16].

In the era of next-generation sequencing, several studies have reported the prevalence of pathogenic variants in multiple genes among breast cancer patients, usually referred and tested based on their premenopausal breast cancer diagnosis and/or relevant family history [17,18,19]. Notably, a limited number of studies focus specifically on young patients, i.e., before the age of 40 years, or very young patients, i.e., before the age of 30 years [20,21,22,23].

We, therefore, sought to investigate the contribution and spectrum of known breast-cancer-associated genes in 267 Greek women diagnosed with very young breast cancer, monitor their clinicopathological characteristics, and assess survival outcomes according to mutation carrier status and time of chemotherapy administration, ultimately aiming at highlighting the causative genetic background of this group of patients and also the importance of early access to this information.

## 2. Materials and Methods

### 2.1. Patient Selection

The study cohort included 267 breast cancer patients, all diagnosed at or before the age of 30 years. Of these, 62 individuals were diagnosed at 26 years, and 205 patients were diagnosed at 26–30 years. Patients have been retrospectively selected solely based on their age from referrals for genetic testing to the Human Molecular Genetics Laboratory of the National Centre for Scientific Research (NCSR) “Demokritos” between the years 1999 and 2021. Written informed consent was obtained from all individuals before genetic testing. This study was approved by the Research Ethics Committee (REC) of NCSR “Demokritos” (43-07/07/2022 and 38-20/06/2023). Detailed family pedigrees were obtained through extended phone interviews. Information concerning the survival of patients was collected up until December 2021. The diagnosis of a second primary tumor, relapse, metastasis, or the occurrence of death was considered an event. Information on family history was extracted from family pedigrees.

### 2.2. Genomic Capture and Massively Parallel Sequencing using the Trusight Cancer Panel and Multiplex Ligation-Dependent Probe Amplification (MLPA)

Germline DNA was extracted from peripheral blood following standard procedures as previously described [24]. All DNA samples were massively parallel sequenced, as mentioned earlier [24]. All pathogenic variants were confirmed by Sanger sequencing. Copy number variants (CNVs) were assessed by MLPA for *BRCA1*, *BRCA2*, and *TP53* since accurate detection is not feasible through our NGS setting. Therefore, SALSA MLPA kits P002, P045, and P056 were used to assess CNVs involving *BRCA1*, *BRCA2*, and *TP53* genes, respectively, following the manufacturer’s instructions (MRC-Holland, Amsterdam, The Netherlands).

### 2.3. Statistical Analysis

Quantitative variables are presented as mean [SD], while categorical variables are presented as percentage (%). Comparisons of categorical variables between groups were performed using Pearson’s chi-square test. A non-parametric Wilcoxon rank-sum test was used to compare the age association between carriers of pathogenic variants per gene. Survival curves were estimated using the Kaplan–Meier method and compared across groups with the log-rank test. Relapse, metastasis, second primary tumor diagnosis, or death were defined as events with a ten-year event-free survival probability. Alive patients were censored at the date of last contact. *p*-values of <0.05 were considered statistically significant.

## 3. Results

### 3.1. Prevalence of Pathogenic Variants

In this very young breast cancer patient cohort (n = 267), the mean [SD] age at diagnosis was 27.49 [2.68] years (range: 18–30 years). Among those, germline pathogenic or likely pathogenic variants (PV/LPV) were identified in 106 patients, resulting in a prevalence of 39.7% distributed in 8 cancer-predisposing genes. *BRCA1* PV/LPVs were the most prevalent, involving 28.1% (75/267) of the patients tested, followed by *TP53* and *BRCA2* PV/LPVs identified in 4.5% (12/267) and 4.1% (11/267) of the patients tested, respectively. PV/LPVs were also identified in *CHEK2* (3), *ATM* (2), *PALB2* (1), *PTEN* (1), and *RAD51C* (1). The overall prevalence of pathogenic variants in *BRCA1*, *BRCA2,* and *TP53* was 36.7% (98/267). In addition, loss-of-function variants were identified in three genes with suspected association with breast cancer, namely, *BRIP1* (2), *NBN* (2), and *BAP1* (1).

**Figure 1:**
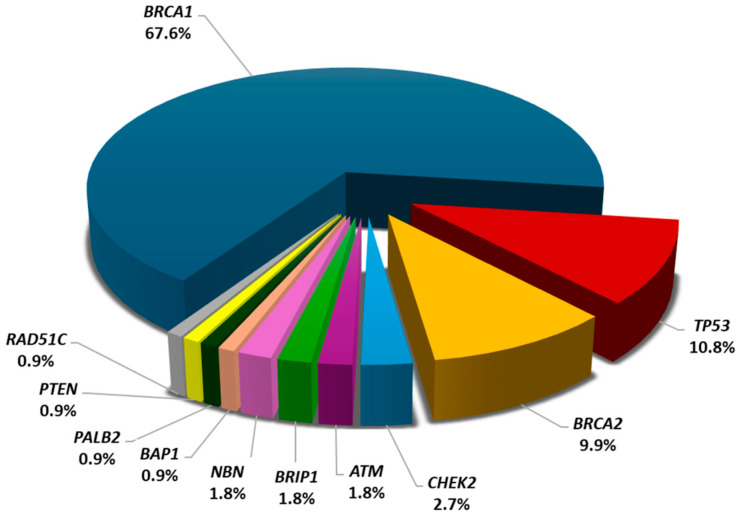
Prevalence and distribution of germline pathogenic variants in known and suspected breast cancer susceptibility genes.

Of the PV/LPVs identified, 7.86% (21/267) involved CNVs. Of these, the vast majority (19/21) involved Greek *BRCA1* and *BRCA2* founders [25,26,27], while one patient carried a deletion of the promoter and exons 1 and 2 of *BRCA1*, and another patient carried a *TP53* deletion encompassing the promoter and the non-coding exon 1. A detailed description of the identified pathogenic variants is summarized in Supplementary Table S1, while their prevalence and distribution are illustrated in Figure 1.

### 3.2. Rates of Pathogenic Variants by Age Group

In a sub-analysis stratified by an extremely young age, the youngest group of patients (≤26 years) showed a significantly higher rate of *TP53* PV/LPVs (12.9%; 8/62) (*p* < 0.01) compared to the older group (26–30 years), where the prevalence of *TP53* PV/LPVs was 1.95% (4/205). *BRCA1/2* pathogenic variants demonstrated the reverse, but not statistically significant, trend with 20.96% (13/62) in the (≤26 years) group, compared to 35.6% (73/205) (*p* = 0.13) in those patients aged 26–30 years.

### 3.3. Associations with Breast Cancer Histopathology

Among the 267 breast cancer patients, 246 (92.2%) had histology reports available. Of the 98 carriers with available histology, ductal carcinomas were the most prevalent (79.6%; 78/98), followed by medullary breast carcinomas (11.3%; 11/98). Interestingly, medullary carcinomas presented a significantly higher frequency in carriers compared to non-carriers (11.3% vs. 2.1%; *p* < 0.05) and were also significantly associated with the presence of *BRCA1* PVs (*p* < 0.05). Similarly, ductal carcinomas were more frequent in *BRCA1* carriers (*p* < 0.05), while tumors of mixed histology were significantly associated with *BRCA2*-positive tumors (*p* < 0.05). Of 234 breast cancer patients with information on tumor grade, 69.3% (162/234) of the breast tumors were grade 3. Carriers had a statistically significant higher rate of grade 3 breast tumors compared to non-carriers (83.7% (82/98) vs. 58.8% (80/136); *p* << 0.01). Similarly, *BRCA1* carriers were more frequently presented with high-grade tumors compared to non-carriers (90% vs. 58.8%; *p* << 0.01, respectively). All histopathological characteristics are summarized in Table 1.

### 3.4. Associations with Breast Cancer Immunohistochemical Subtypes

Hormone receptor (HR) and HER2 status were assessed from immunohistochemical (IHC) reports for 256 breast cancer patients (of which 102 are carriers). Among these, 28.7% (75/256) involved (HR+), HER2(−) diagnoses, 15.1% (39/256) (HR+), HER2(+) diagnoses, 8.5% (22/256) (HR−), HER2(+) diagnoses, and 38.8% (100/256) involved triple-negative breast cancer (TNBC), including basal-like type.

In carriers, the histopathology of breast tumors involved 19.6% (20/102) (HR+), HER2(−), 9.8% (10/102) (HR+), HER2(+), 4.9% (5/102) (HR−), HER2(+), and 62.8% (64/102) TNBC diagnoses.

The TNBC breast cancer subtype was statistically significantly more frequent in carriers compared to non-carriers (62.8% vs. 23.4%, *p* << 0.01). TNBC was the most frequent breast cancer immunophenotype in *BRCA1* carriers (75.6%). Interestingly, TNBC was diagnosed in 44.5% and 25% of *BRCA2* and *TP53* carriers, respectively. All data are summarized in Table 1.

### 3.5. Associations with Family History

Information on family history was available for 247 out of the 267 patients in this study. Positive family history for any type of cancer with suspected hereditary etiology was significantly more frequent in carriers compared to non-carriers (74.8% vs. 35.2%; *p* < 0.05). Specifically, 76.1% of *BRCA1* carriers reported positive family history, which was statistically significantly higher compared to non-carriers (76.1% vs. 35.2%; *p* < 0.05). The same observation on the family history involved *BRCA2* and *TP53* carriers when compared to non-carriers (77.8% vs. 35.2%; *p* < 0.05 and 75% vs. 35.2%; *p* < 0.05, respectively).

However, a little over one-fourth (25/99) of carriers did not report any family history of cancer. This was further investigated by monitoring plausible limiting factors. Herein, 28% (7/25) of carriers with no reported family history had a predominance of male blood relatives, 28% (7/25) had a small family structure, and one case had both of these factors. Furthermore, one patient was found to carry the PV in mosaicism, while two others had a de novo PV. Paternally inherited PVs were reported in 12% (3/25) while limited information due to loss of contact with their family relatives was reported in 16% (4/25).

### 3.6. Associations with Age at First Breast Cancer Diagnosis

Women who carried *TP53* PVs were diagnosed with breast cancer at a statistically significant younger age compared to non-carriers (mean [SD] age: 24.75 [3.00] vs. 27.31 [2.75] years, *p* << 0.01, respectively). However, age at breast cancer diagnosis was not significantly associated with *BRCA1*, *BRCA2,* or other gene carrier status. Notably, *TP53* carriers were diagnosed with breast cancer earlier when compared to *BRCA1* (24.75 y [3.00] vs. 27.96 y [2.41], *p* << 0.01) or *BRCA2* carriers (24.75 y [3.00] vs. 28.36 [1.77], *p* << 0.01).

### 3.7. Families and Individuals with TP53 PVs and Association with Li–Fraumeni Syndrome

A total of 4.5% of the women included in this study carried *TP53* PVs, which are known to be associated with Li–Fraumeni syndrome (LFS). Among these *TP53* carriers, 75% (9/12) fulfilled the classic Chompret criteria for *TP53* genetic testing [28], having a typical personal and/or family history of LFS. Among the three patients who did not meet the classic Chompret criteria, one proved to carry the PV in mosaicism, and two had a possibly de novo pathogenic variant [15]. Notably, 58.3% (7/12) of *TP53* carriers developed multiple tumors. Breast tumors presented in half of *TP53* carriers were HER-2 positive.

### 3.8. Patient Outcomes

**Figure 2:**
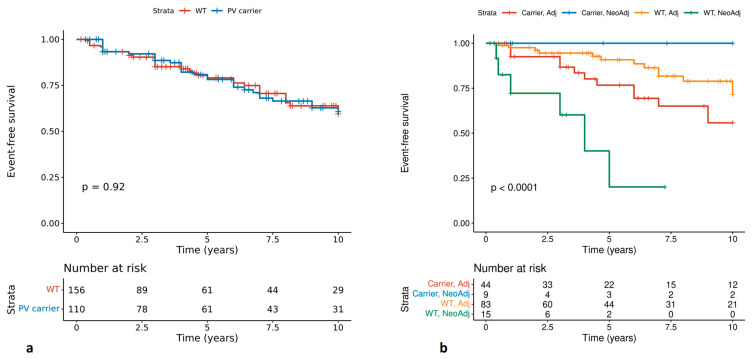
Event-free survival and neo-adjuvant chemotherapy. (**a**) Comparison among carriers and non-carriers of pathogenic variants; (**b**) Considering the time chemotherapy was undertaken (neoadjuvant vs. adjuvant setting).

The event-free survival (EFS) of patients has been assessed. Two patients were de novo metastatic and were not included in the analysis. The median follow-up of patients from diagnosis until death or last contact was 62 months (ranging from 6 to 439 months). There was no statistical significance on EFS when carriers and non-carriers were compared (Figure 2a). In a sub-analysis, we assessed whether carrying a PV on a specific gene had an impact on survival. Although this analysis did not reach statistical significance, *TP53* carriers had the worst EFS (survival frequency: 0.364; 95% CI, 0.132–1; *p* = 0.38), compared to carriers in other cancer-predisposing genes (survival frequency: 0.667; 95% CI, 0.402–1, *p* = 0.38) (Supplementary Figure S1).

Survival was also assessed based on the time chemotherapy was undertaken (neoadjuvant vs. adjuvant setting). This analysis included 151 patients based on available information. Carriers who received neoadjuvant chemotherapy showed improved 10-year EFS (no events recorded; *p* < 0.0001), compared to patients that received adjuvant therapy irrespectively of their carrier status [carriers: 10-year EFS probability, 0.558; 95% CI, 0.396–0.7844; *p* < 0.0001, non-carriers: 10-year EFS probability, 0.714; 95% CI, 0.583–0.874; *p* < 0.0001] (Figure 2b)**.**

## 4. Discussion

In this study, we investigated the contribution and spectrum of known breast-cancer-associated genes in 267 Greek women diagnosed with breast cancer ≤30 years. Subsequently, we evaluated the correlation of our data with patient outcomes and clinico-histopathological characteristics.

Herein, a significant fraction, i.e., 39.7%, of the patients tested were found to carry germline PVs in 8 distinct breast cancer-predisposing genes. The three highly-penetrant genes *BRCA1*, *BRCA2,* and *TP53* attained the vast majority (36.7%) of PVs identified, with *BRCA1* being the most prevalent. Notably, *TP53* PVs, identified in 4.5% of the patients tested, were the second most frequently observed herein, following *BRCA1* PVs, constituting a relatively high rate compared to other studies [23]. Moreover, this observation is in contrast with previous studies on young breast cancer patients, where the second most prevalent gene identified was *BRCA2*, exhibiting rates comparable to those of *BRCA1* [22,29,30]. This can be possibly explained by the characteristic Greek genetic makeup, which is strongly influenced by founder effects affecting the *BRCA1* gene [31]. Therefore, *BRCA2* PVs are found at a notably lower frequency compared to other Caucasian populations [32].

The detection rate of PVs in the other five genes (*ATM*, *CHEK2*, *PALB2*, *PTEN*, and *RAD51C*) with a clear association with breast cancer susceptibility was only 3%, indicating that PVs in these genes are relatively rare in early-onset breast cancer patients and that the clinical benefit of analyzing these genes in this group of patients is limited, in line with other studies [23,30]. This can be justified by the fact that these genes exhibit medium penetrance and are characterized by an older median age of onset for breast cancer [7].

Furthermore, our study revealed that one-fourth of *TP53* carriers did not meet the classic Chompret criteria [28]. Consequently, a potential diagnosis of LFS might have been overlooked if a genetic analysis was solely restricted to *BRCA1* and *BRCA2* testing. This oversight could significantly impact appropriate patient clinical management since individuals with LFS have an increased lifetime risk of developing additional malignancies and are characterized by increased radiosensitivity [11,15,33]. These distinctive characteristics of LFS patients necessitate the adoption of specialized surveillance protocols, emphasizing the imperative to minimize exposure to radiation, whether for therapeutic or monitoring purposes. Taking into consideration the high percentage of de novo cases in LFS, the revised Chompret criteria [15] have included breast cancer under 31 years as a standalone criterion for consideration of *TP53* testing. However, this has not yet become common practice in several countries, mainly due to financial restrictions and a lack of specialized genetics clinics. Our results emphasize the need for the inclusion of *TP53* in the genetic testing algorithm, in addition to *BRCA1* and *BRCA2* analysis, in all women diagnosed with breast cancer at young onset [34]. This is of high importance in countries like Greece, where the National Insurance Policies reimburse the cost for genetic analysis of *BRCA1* and *BRCA2* only.

The occurrence of *BRCA1* and *BRCA2* PVs was less frequent in the extreme age group of our cohort (≤26 years), whereas *TP53* PVs were notably more prevalent compared to the patient group with diagnoses between 26 and 30 years. This finding aligns with the study conducted by Evans et al. [30]. The observed trend underscores the importance of conducting *TP53* testing as close to their diagnosis as possible in very young women with breast cancer. Given the rarity of breast cancer diagnoses before the age of 26 and the limited availability of high-volume centers specializing in their management, healthcare professionals handling these cases must be cognizant of this critical aspect. Due to the heightened risk of contralateral breast cancer and the increased radiosensitivity in these women, timely discussions regarding risk-reducing mastectomy are imperative.

Histopathological and family history correlations in this very young group of breast cancer patients mainly emphasized previously well-established associations but also revealed some interesting new aspects. Triple-negative immunophenotype, high-grade tumors, and medullary subtype were all strongly associated with *BRCA1* PVs in our study, as previously shown [35,36,37,38,39]. Interestingly, we noticed that breast tumors with mixed histology were mainly *BRCA2*-related. It is worth highlighting that high-grade disease alone was the most uniform predictor of carrier status, irrespective of the gene involved, as 83.7% of carriers had grade 3 tumors. Surprisingly, this correlation was a stronger predictor than family history, which was reported as positive in 74.8% of the carriers.

Survival rates of breast cancer patients diagnosed before the age of 35 years are poor [40,41,42,43,44]. It is not surprising that, herein, women with *TP53* PVs showed the worst survival outcomes compared with carriers of other predisposing genes. This observation can be attributed to the more aggressive tumor biology and comorbidities of *TP53* carriers. More aggressive tumor biology overall is prominent in the cohort studied herein, with 69.3% presenting with high-grade tumors and 38.8% with triple-negative tumors, justifying the poor survival rates.

The EFS rates did not exhibit discernible differences between carriers and non-carriers, consistent with findings from previous studies [1]. On the contrary, a noteworthy observation emerged: carriers who underwent neoadjuvant chemotherapy demonstrated a statistically significant improvement in EFS compared to patients receiving adjuvant chemotherapy, irrespective of carrier status. It is important to interpret these findings cautiously, as the sample size is relatively small, and additional studies are warranted to validate these observations. Moreover, this observation might be restricted to this very young group of patients. However, it is noteworthy that earlier research has linked neoadjuvant chemotherapy to a more favorable pathological response in gene carriers [45,46]. If substantiated, the prompt identification of a patient’s carrier status within this age group becomes crucial, given the potential clinical benefits associated with neoadjuvant therapy.

This study is subject to certain limitations. Obtaining access to histopathology reports, detailed family history, and current vital status proved unfeasible for all participants. Additionally, while this study encompasses a relatively substantial number of patients, given the stringent age criteria, the sample size remains constrained for robust statistical associations. Despite the absence of specific selection criteria aside from a young age, biases may exist in patient referral and consent for genetic testing. Moreover, detailed data on the exact chemotherapy treatment plans or regimens used were not monitored. It is possible that the observed survival benefit might be influenced by treatment and/or regimens that have been modified over time. Lastly, the data presented herein originate from a specific population with a demonstrated genetic makeup; thus, their representativeness for other populations may be limited.

The strength of this study lies in the relatively large number of very young breast cancer patients, affording the unique opportunity to meticulously examine gene spectra, establish clinicopathological associations, and analyze clinical outcomes. Given the limited number of studies that specifically address this patient group, the extrapolated data presented herein can significantly contribute to the enhanced management of this distinctive group of patients.

## 5. Conclusions

In summary, our findings reveal a notable prevalence of pathogenic variants, predominantly within three highly penetrant breast cancer susceptibility genes, among women diagnosed with very young breast cancer. The elevated occurrence of *TP53* pathogenic variants underscores the importance of incorporating this gene in the genetic analysis offered to this specific patient group, as relying solely on *BRCA1* and *BRCA2* testing proves inadequate. Consequently, timely access to genetic test results, along with appropriate genetic counseling, particularly for very young breast cancer patients, facilitates tailored clinical management and offers essential opportunities for informed decision-making regarding prevention strategies and family planning.

## Figures and Tables

**Table 1:** Patients’ clinicopathological characteristics.

	Cases	Carriers	*BRCA1*	*BRCA2*	*TP53*
**Age of onset (y)**					
Mean [SD]	27.49 [2.68]	27.77 [2.54]	27.96 [2.41]	28.36 [1.77]	24.75 [3] *
Min–Max	17–31				
**Histology**					
Informative	246	98	70	9	12
Ductal (N=), (n%)	208 (84.6)	78 (79.6)	54 (77.1) *	6 (66.7)	11 (91.6)
Lobular (N=), (n%)	7 (2.8)	1 (1)	1 (1.4)	0 (0)	0 (0)
Medullary (N=), (n%)	14 (5.7)	11 (11.3) *	9 (12.9) *	1 (11.1)	1 (8.4)
Mixed (N=), (n%)	7 (2.8)	5 (5.1)	3 (4.3)	2 (22.2) *	0 (0)
DCIS (N=), (n%)	6 (2.5)	1 (1)	1 (1.4)	0 (0)	0 (0)
Other (N=), (n%)	4 (1.6)	2 (2)	2 (2.9)	0 (0)	0 (0)
**Grade**					
Informative	234	98	70	9	12
I (N=), (n%)	12 (5.1)	2 (2.1)	1 (1.4)	0 (0)	1 (8.3)
II (N=), (n%)	60 (25.6)	14 (14.2)	6 (8.6)	3 (33.3)	3 (25)
III (N=), (n%)	162 (69.3)	82 (83.7) *	63 (90) *	6 (66.7)	8 (66.7)
**SubTypes**					
Informative	256	102	74	9	12
HR+, HER2- (N=), (n%)	75 (28.7)	20 (19.6)	11 (14.9)	1 (11.1)	3 (25)
HR+, HER2+ (N=), (n%)	39 (15.1)	10 (9.8)	2 (2.7)	3 (33.3)	3 (25)
HR-, HER2+ (N=), (n%)	22 (8.5)	5 (4.9)	2 (2.7)	0 (0)	3 (25)
TNBC (N=), (n%)	100 (38.8)	64 (62.8) *	56 (75.6) *	4 (44.5)	3 (25)
Other (N=), (n%)	20 (8.9)	3 (2.9)	3 (4.1)	1 (11.1)	0 (0)
**Family History**					
Informative	247	99	71 *	9 *	12 *
Yes	128	74 (74.8) *	54 (76.1) *	7 (77.8) *	9 (75) *
No	122	25 (25.2)	17 (23.9)	2 (22.2)	3 (25)

* statistically significant associations.

## Data Availability

All data that support the findings of our study are available upon reasonable request.

## Supplementary Materials

The following supporting information can be downloaded at: https://www.mdpi.com/article/10.3390/cancers16132368/s1, Table S1: Description of germline pathogenic variants identified in the study; Figure S1: Impact of the presence of pathogenic variants on the EFS in patients with early-onset breast cancer.
