# Supplementary material for: Genetic Testing of Breast Cancer Patients with Very Early-Onset Breast Cancer (≤30 Years) Yields a High Rate of Germline Pathogenic Variants, Mainly in the BRCA1, TP53, and BRCA2 Genes
Source: Cancers (Basel). 2024 Jun 27;16(13):2368. doi: 10.3390/cancers16132368 (PMC11240773; doi:10.3390/cancers16132368)

Supplementary Materials

Legends

Table S1. Description of germline pathogenic variants identified in the study.

Figure S1. Impact of the presence of pathogenic variants on the EFS in patients with early onset breast cancer.

Supplementary Table S1. Description of germline pathogenic variants identified in the study

Patient ID	Gene	DNA variation	Predicted Protein consequence
**F3228**	*ATM*	c.5979_5983del	p.(Ser1993ArgfsTer23)
**F3068**	*ATM*	c.8122G>A	p.(Asp2708Asn)
**F124**	*BAP1*	c.1203T>G	p.(Tyr401Ter)
**F2394**	*BRCA1*	g.(41267773_41276097)_(41278204_41282088)del	p.(?)
**F1472**	*BRCA1*	c.302-2A>G	p.?
**F1238**	*BRCA1*	c.1059G>A	p.(Trp353Ter)
**F315**	*BRCA1*	c.1504_1508del	p.(Leu502AlafsTer2)
**F1235**	*BRCA1*	c.1521_1531del	p.(Thr509SerfsTer3)
**F130**	*BRCA1*	c.1885A>T	p.(Arg629Ter)
**F2999**	*BRCA1*	c.2019del	p.(Glu673AspfsTer28)
**F3300**	*BRCA1*	c.2269del	p.(Val757PhefsTer8)
**F796**	*BRCA1*	c.2963C>A	p.(Ser988Ter)
**F3961**	*BRCA1*	c.3157dup	p.(Glu1053GlyfsTer7)
**F483**	*BRCA1*	c.3375_3376del	p.(Pro1126IlefsTer6)
**F1144**	*BRCA1*	c.3375_3376del	p.(Pro1126IlefsTer6)
**F230**	*BRCA1*	c.3607C>T	p.(Arg1203Ter)
**F430a**	*BRCA1*	c.3607C>T	p.(Arg1203Ter)
**F1789**	*BRCA1*	c.3607C>T	p.(Arg1203Ter)
**F820**	*BRCA1*	c.3624del	p.(Lys1208AsnfsTer1)
**F558**	*BRCA1*	c.3700_3704del	p.(Val1234GlnfsTer8)
**F3597**	*BRCA1*	c.3700_3704del	p.(Val1234GlnfsTer8)
**F3448**	*BRCA1*	c.4096+1G>A	p.Ala224_Leu1365del
**F1675**	*BRCA1*	c.4327C>T	p.(Arg1443Ter)
**F2099**	*BRCA1*	c.4327C>T	p.(Arg1443Ter)
**F678**	*BRCA1*	c.4576G>T	p.(Glu1526Ter)
**F3790**	*BRCA1*	c.5098A>C	p.(Thr1700Pro)
**F470**	*BRCA1*	c.5152+1G>A	p.Asp1692_Trp1718del
**F276**	*BRCA1*	c.5212G>A	p.(Gly1738Arg)
**F707a**	*BRCA1*	c.5212G>A	p.(Gly1738Arg)
**F739**	*BRCA1*	c.5212G>A	p.(Gly1738Arg)
**F1539**	*BRCA1*	c.5212G>A	p.(Gly1738Arg)
**F1652**	*BRCA1*	c.5212G>A	p.(Gly1738Arg)
**F3658**	*BRCA1*	c.5212G>A	p.(Gly1738Arg)
**F3800**	*BRCA1*	c.5212G>A	p.(Gly1738Arg)
**F1420**	*BRCA1*	c.5246C>G	p.(Pro1749Arg)
**F298**	*BRCA1*	c.5251C>T	p.(Arg1751Ter)
**F1198**	*BRCA1*	c.5251C>T	p.(Arg1751Ter)
**F1687**	*BRCA1*	c.5251C>T	p.(Arg1751Ter)
**F2229a**	*BRCA1*	c.5251C>T	p.(Arg1751Ter)
**F2754**	*BRCA1*	c.5251C>T	p.(Arg1751Ter)
**F3450**	*BRCA1*	c.5251C>T	p.(Arg1751Ter)
**F582**	*BRCA1*	c.5256_5277+3179del	p.Ala1752_Lys1759del
**F3227**	*BRCA1*	c.5256_5277+3179del	p.Ala1752_Lys1759del
**F371**	*BRCA1*	c.5266dup	p.(Gln1756ProfsTer74)

**F655**	*BRCA1*	c.5266dup	p.(Gln1756ProfsTer74)
**F777**	*BRCA1*	c.5266dup	p.(Gln1756ProfsTer74)
**F933**	*BRCA1*	c.5266dup	p.(Gln1756ProfsTer74)
**F1792**	*BRCA1*	c.5266dup	p.(Gln1756ProfsTer74)
**F1862**	*BRCA1*	c.5266dup	p.(Gln1756ProfsTer74)
**F2590**	*BRCA1*	c.5266dup	p.(Gln1756ProfsTer74)
**F2714**	*BRCA1*	c.5266dup	p.(Gln1756ProfsTer74)
**F3373a**	*BRCA1*	c.5266dup	p.(Gln1756ProfsTer74)
**F3432**	*BRCA1*	c.5266dup	p.(Gln1756ProfsTer74)
**F3442**	*BRCA1*	c.5266dup	p.(Gln1756ProfsTer74)
**F3705**	*BRCA1*	c.5266dup	p.(Gln1756ProfsTer74)
**F1343**	*BRCA1*	c.5278-1G>T	p.Ile1760_Asp1778del
**F062**	*BRCA1*	c.5407-754_5592+8273del	p.(Gly1803_Tyr1863del)
**F416**	*BRCA1*	c.5407-754_5592+8273del	p.(Gly1803_Tyr1863del)
**F686**	*BRCA1*	c.5407-754_5592+8273del	p.(Gly1803_Tyr1863del)
**F851**	*BRCA1*	c.5407-754_5592+8273del	p.(Gly1803_Tyr1863del)
**F976**	*BRCA1*	c.5407-754_5592+8273del	p.(Gly1803_Tyr1863del)
**F2740**	*BRCA1*	c.5407-754_5592+8273del	p.(Gly1803_Tyr1863del)
**F2887**	*BRCA1*	c.5407-754_5592+8273del	p.(Gly1803_Tyr1863del)
**F2911**	*BRCA1*	c.5407-754_5592+8273del	p.(Gly1803_Tyr1863del)
**F3222**	*BRCA1*	c.5407-754_5592+8273del	p.(Gly1803_Tyr1863del)
**F500**	*BRCA1*	c.5431C>T	p.(Gln1811Ter)
**F757**	*BRCA1*	c.5431C>T	p.(Gln1811Ter)
**F178**	*BRCA1*	c.5467G>A	p.(Gly1803_Ala1813del)
**F240**	*BRCA1*	g.79398_83825del	p.0
**F332**	*BRCA1*	g.79398_83825del	p.0
**F382**	*BRCA1*	g.79398_83825del	p.0
**F567**	*BRCA1*	g.79398_83825del	p.0
**F736**	*BRCA1*	g.79398_83825del	p.0
**F1059**	*BRCA1*	g.79398_83825del	p.0
**F782**	*BRCA1*	c.5492del	p.(Pro1831Leufs)
**F1460**	*BRCA1*	c.5497G>A	p.(Val1833Met)
**F3367**	*BRCA1*	c.5497G>A	p.(Val1833Met)
**F135**	*BRCA2*	c.4409_4410del	p.(Ile1470LysfsTer11)
**F967**	*BRCA2*	c.4415_4418del	p.(Lys1472ThrfsTer6)
**F2264**	*BRCA2*	c.5645C>A	p.(Ser1882Ter)
**F3508**	*BRCA2*	c.6490C>T	p.(Gln2164Ter)
**F903**	*BRCA2*	c.6842-2675_7008-5558del	p.(Gly2281_Arg2336del)
**F943a**	*BRCA2*	c.6842-2675_7008-5558del	p.(Gly2281_Arg2336del)
**F852**	*BRCA2*	c.6941del	p.(Thr2314LysfsTer2)
**F465**	*BRCA2*	c.8993_9024del	p.(Ser2998IlefsTer9)
**F2420**	*BRCA2*	c.9246_9247insA	p.(Thr3085AsnfsTer26)
**F3873**	*BRCA2*	c.9018C>A	p.(Tyr3006Ter)
**F160**	*BRCA2*	c.9382 C>T	p.(Arg3128Ter)
**F3252**	*BRIP1*	c.984_990del	p.(Phe328LeufsTer8)
**F1473**	*BRIP1*	c.2947del	p.(Ile983LeufsTer2)
**F1484**	*CHEK2*	c.549G>C	p.(Leu183Phe)
**F2839**	*CHEK2*	c.1188del	p.(Val397PhefsTer17)

**F3710a**	*CHEK2*	c.592+3A>T	p.(Val148PhefsTer7)
**F1487**	*NBN*	c.2140C>T	p.(Arg714Ter)
**F3449**	*NBN*	c.2140C>T	p.(Arg714Ter)
**F924**	*PALB2*	c.1591_1600del	p.(Pro532AlafsTer26)
**F975**	*PTEN*	c.424del	p.(Arg142GlyfsTer5)
**F2615**	*RAD51C*	c.577C>T	p.(Arg193Ter)
**F1686**	*TP53*	g.(7579941_7590694)_(7590868_?)del	p.(?)
**F920**	*TP53*	c.324_327dup	p.(Arg110PhefsTer40)
**F3685**	*TP53*	c.329_330insGTTTCCG	p.(Leu111PhefsTer40)
**F329**	*TP53*	c.375G>A	p.(Ser33_Thr125del)
**F1995**	*TP53*	c.559+2T>C	p.?
**F2533**	*TP53*	c.560G>T	p.(Gly187Val)
**F3364**	*TP53*	c.570del	p.(Pro191LeufsTer56)
**F1966**	*TP53*	c.578A>T	p.(His193Leu)
**F1212**	*TP53*	c.637C>T	p.(Arg213Ter)
**F3293**	*TP53*	c.658T>C	p.(Tyr220His)
**F747**	*TP53*	c.743G>A	p.(Arg248Gln)
**F1049**	*TP53*	c.825T>A	p.(Cys275Ter)

Figure S1. Impact of the presence of pathogenic variants on the EFS in patients with early onset breast cancer.

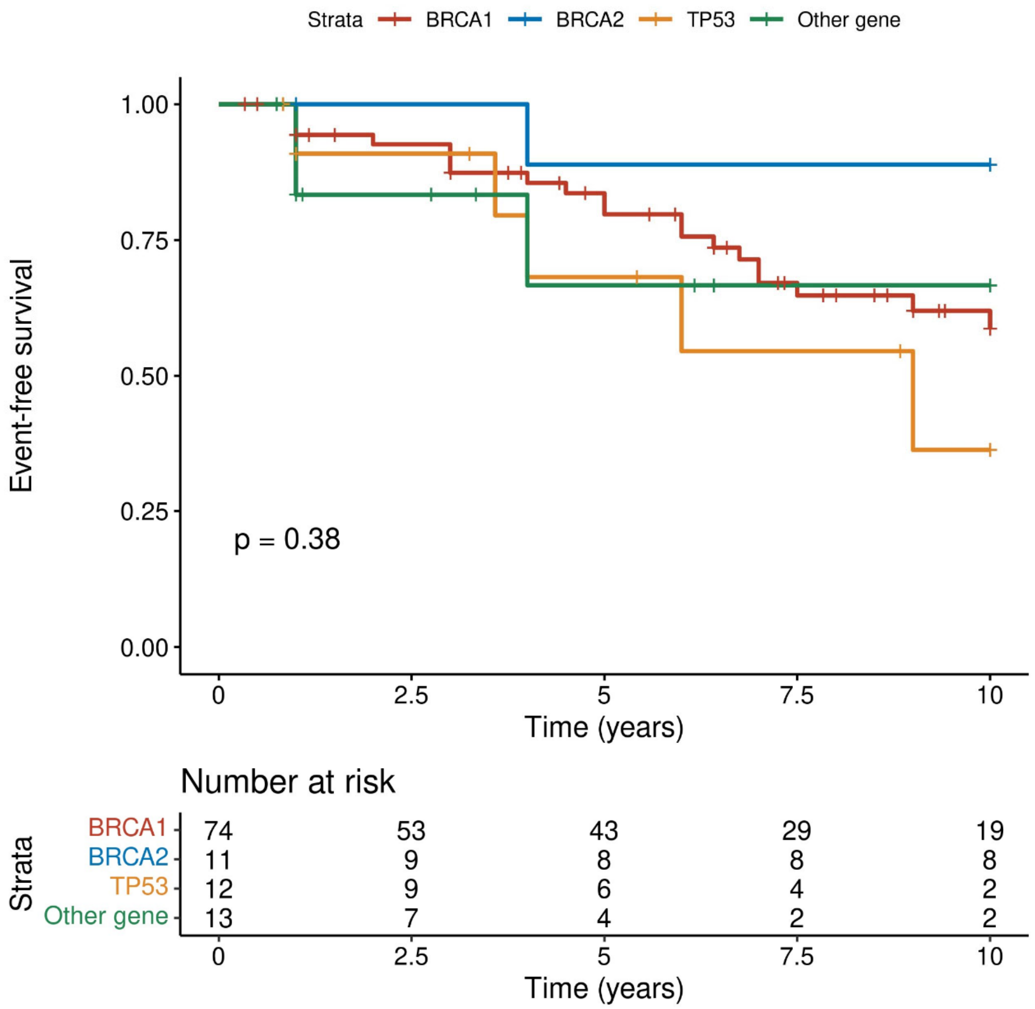

Supplement: Supplementary file 1 [file cancers-16-02368-s001.zip › cancers-3067616-supplementary.pdf]
